# Data Collection in Times of Pandemic: A Self-Study and Revisit of Research Practices During a Crisis

**DOI:** 10.1177/21582440231160698

**Published:** 2023-03-27

**Authors:** Chinaza Uleanya, Ke Yu

**Affiliations:** 1 University of Johannesburg Faculty of Education, South Africa

**Keywords:** COVID-19, data collection, remote data collection, self-study

## Abstract

COVID-19 as a global pandemic has greatly disrupted research, not only in terms of the practicality of research activities such as data collection, but also in data quality. Using self-study in form of duoethnography method for reflecting on research practice, this article reviews and reflects on the practices of remote data collection during the pandemic and further revisits additional issues brought about by these practices and concerns. One key observation from this self-study is the prevalence of practical challenges, particularly those related to participant access, that overshadows the potential advantages of remote data collection as well as other challenges. This challenge results in researchers’ reduced control of the research process and also a requirement for more flexibility, greater sensitivity toward the participants and research skills for the researchers. We also observe greater conflation of quantitative and qualitative data collection and the emergence of triangulation as the main strategy to offset potential threats to data quality. This article concludes by calling for more discussions on several areas that feature scarce discussion in literature, including potential rhetoric importance assigned to data collection, adequacy of triangulation to safeguard data quality, and the potential difference between COVID-19’s impact on quantitative and qualitative research.

## Introduction

Data collection in times of crisis is challenging (Bratcher, 2020; Qadir, 2016). However, Jansen, a technical advisor for the United Nations Population Fund reminds that “no situation justifies unethical, unreliable and un-actionable data collection exercises” (p. 1). Like other pandemics, COVID-19 has greatly disrupted the research production although its scale and impact are unprecedented in recent history. The first set of COVID’s impact relates to change in priority. For most universities—the backbone of research production entities—the common response and focus since the onset of the pandemic has been the transition toward emergency online teaching (Uleanya et al., 2021). Although research is where Humboldtian universities’ prestige lie, teaching remains one fundamental and core purpose of the universities. For academics with teaching responsibilities, this change to complete online teaching—accelerating online or blended experiments for some and a complete shift from face-to-face to online for others—has been disorienting and overwhelming (Jandrić et al., 2020; Merrill, 2020; Volschenk et al., 2021). Like other professionals, many academics also face the challenges of creating space (both physical and mental) when home and work are increasingly fused. On the research front, COVID-19 related research topics, especially those related to understanding the nature of the pandemic, its diagnosis or retreatment naturally assume priority (e.g., Mackenzie et al., 2021). Journals across the fields such as *Autism*, *Environment Systems and Decisions, Food Security, International Journal of Educational Development*, Irish Educational Studies, Nursing Education Perspectives, Psychological Assessment, *PLOS ONE*, etc. all set aside COVID-19 related special volumes.

The second set of impacts from COVID-19 relates to availability. The ever-changing situation of COVID-19, as well as the resulting uncertainty, escalates anxieties, and stress levels for all professions, including potential participants, many of whom becoming less available or willing to avail themselves to research (Bratcher, 2020). With social distance regulations and the expectation of research doing no harm, some ethics committees and other authorities are also less willing to approve or grant research access; some resort to imposing stricter terms on research topics or study populations (Gentili & Cristea, 2020). As economics flounders, funding priorities are reset and overall funds available dwindle. Travel was dramatically reduced if not grinding to a halt; so are some collaborations. Some researchers moved to secondary data analysis to bypass data collection; others persevered. Research activities that can be moved online did so. For topics or methods which render themselves not viable to remote platforms, for example, observations, certain behavioural studies, hospital visits, or neuroimaging studies (in confined space), studies are halted or paused (van Dorn, 2020). For projects commenced before COVID-19, many projects were delayed, put on hold, or resumed with revised data collection methods. One group that starts new research projects during COVID-19 is coursework postgraduate students who had completed their coursework and are under pressure to continue and complete their qualifications within a given time frame (Paula, 2020; Persky et al., 2020).

This article reviews and reflects the practices of remote data collection during the pandemic and further revisits additional issues brought about by these practices and concerns. This is to respond to a literature gap Chatha and Bretz (2020) identify where “much has been written about the challenges of moving from face-to-face to remote teaching and learning…comparatively little has been written about how human subjects research…adapted to continue during the pandemic” (p. 4196). One unique feature of this article is its methodology, self-study. Self-study is a methodology increasingly used in reflecting teaching practice, but remains rarely used to reflect research practice. Another unusual feature of this article is the organization of its examination and reflection: instead of organizing the reflection and discussion per the qualitative and quantitative research approach common in published research reflections (e.g., Sy et al., 2020; Torrentira, 2020), the discussion in this article is organized along the data collection process itself (similar to Hensen et al., 2021). Although when applicable, we also separated discussions for quantitative and qualitative in our reflection, we increasingly are aware that many issues are no longer unique to one research approach. Although we have tried to distill any specific impact on either of the research approach, our experience, preceding COVID-19 and more cemented during COVID-19, indicates that the boundary between the approaches is increasingly blurred. Since both authors research within education and social science, our experience and reflection are limited to these domains. The article concludes with implications of our discussion for the broader research methodology community. The article is organized as follows: after reviewing relevant literature on innovative data collection before and during COVID-19, it introduces and presents the methodology used for this article. Thereafter, reflection and critical dialogues on researcher identity and impact on research, sampling strategy and participant recruitment, data collection platforms and instrument, and ethical considerations and practices followed. The article concludes with recommendations for both research methodology broadly and also data collection practice more specifically.

### Literature Review

Contradictions shroud the importance of data collection in empirical studies. On one hand, data collection is generally accepted as a critical step in any empirical study since data determines the outcome and quality of a study (Bhasin, 2021; Kabir, 2016): garbage in and garbage out. Data collection is more crucial in qualitative studies or studies that involve a number of phases (e.g., a sequential mix method study) when data already collected might alter subsequent research decisions (e.g., modification or creation of instrument). Data collection also helps identify further areas of research, refine existing research questions or research instruments (Bhandari, 2021). Data should not only be information-rich but also relevant, reliable, and trustworthy (AnswerMiner, 2018; Bhasin, 2021; Davis, 2021). On the other hand, data collection is often the *first* item to be outsourced in large projects or when research funding is available. Fieldworkers who are involved only in collecting data but not other phases in research (e.g., research analysis) are routinely excluded from the author list but relegated to the acknowledgment page. Although the influence of the skills of the fieldworkers on the research process and output is recognized, data collection is often outsourced to enable researchers to cope with the increased workload during the research (Šegan & Verbič, 2017). The typical reason cited for this practice is that data collection is “time-consuming” (HabileData, n.d, 1), implying that researchers’ valuable time should be devoted to more important work. This again reinforces the low status attached to data collection. Limited literature, however, discusses or debates this.

The time needed to collect data, data collection skills, and data quality are all considerations when data collection plans are conceived. Most specifically, many relevant decisions often require a balance between practicality (researcher time or skills, interest, and availability on the part of the participants, and available technology) and data quality. This balancing act is what this reflection and discussion in the review primarily focus on.

### Innovation in Data Collection Before COVID-19

Researchers have experimented with remote or other innovative ways to collect data before the outbreak of COVID-19, for example, during crises such as SARS, chronic warfare, or other challenging context for data collection (Korinek et al., 2019; Person et al., 2004). The work of Jerbi (2021) highlights one key principle when exploring alternative ways to collect data: being flexible and open to change plans. One main challenge and often the aim for innovative data collection experimentations is to enhance participants’ accessibility, so that the realized sample size is larger, generalizability greater, and research findings more reliable (Howard, 2019). This is often achieved through broadening access and increased participant pool, often via accessing a larger geographical area or reaching previously harder to reach population (Archibald et al., 2019; Dickinson et al., 2019; Morse, 2015). Remote data collection can also reduce costs (e.g., for printing questionnaires or travel) and assist other logistical matters such as carrying bulky questionnaires, speeding up data entry, cleaning, and process time for data analysis (Hensen et al., 2021; Sy et al., 2020). In addition, data entry rules can be imposed to reduce human error (Dickinson et al., 2019).

Comparison of costs, logistics, participants’ experience, and data quality between remote and face-to-face data collection remains limited (Daniels et al., 2019) however. No consensus regarding which one is better has emerged too. While the disparity between face-to-face and remote data collection is found in some studies (e.g., Germine et al., 2012; Krouwel et al., 2019), “very few differences in the richness of data collected” (Daniels et al., 2019, p. 2) is concluded in others where “audio-visual environment is generally seen as closely align with the face-to-face environment” (Matthews et al., 2018. Similar finding from Dickinson et al., 2019; Trate et al., 2020).

Remote data collection tools can be executed online or through other remote platforms (Archibald et al., 2019; Daniels et al., 2019; Kite & Phongsavan, 2017; Lobe, 2017; Lobe et al., 2020; Matthews et al., 2018) although some scholars (e.g., Archibald et al., 2019; Salmons, 2012) further differentiate and categorize in terms of whether such data collection is synchronized (exchange in real-time, e.g., through a chat room, Zoom, telephone, WhatsApp call, video conference applications), near-Synchronous (near-immediate post and response) for example, Short Message Service (SMS) and WhatsApp messaging, or asynchronized (time lapse between message and response, e.g., through email, instant messaging, Short Message Service (SMS), and discussion forum). However, like the comparison between remote and face-to-face data collection, the comparison of data collected through synchronized or asynchronized means remains limited too. Among the few that do, Brüggen and Willems (2009) suggests that asynchronous data collection produces high depth and breadth data.

For surveys, Google form, Survey Monkey, and Qualtrics (some with basic analysis functions), mobile phone surveys, etc., are often used. For interviews and focus groups, video conference platforms (e.g., Zoom, Microsoft Teams, or Skype) are usually preferred (Yardley, 2020), although other interactive voice responses, instant messaging services (such as Whatsapp and WeChat), other means without visual (e.g., telephone, text-based, or audio-only interactions) are also routinely used (Abrams et al., 2015). Among these, Zoom has clearly emerged as one of the most popular platforms before and during COVID-19 (Archibald et al., 2019; Daniels et al., 2019; Lobe et al., 2020). Other online data collection platforms that have been experimented with include Google Handouts or other electronic survey packages (*FIlemaker* in Dickinson et al., 2019) and Whatsapp survey (Chen et al., 2020). Some researchers have also experimented with participants’ self-collected data, for example, through reflective journals or audio diaries (Lupton, 2020; Mupambireyi & Bernays, 2019), photovoice (using photography to capture lived experiences, Copes et al., 2018; Sutton-Brown, 2014). Other researchers forego data collection altogether and change to secondary data analysis from the existing data repository (Kozinets, 2019; United Nations High Commissioner for Refugees [UNHCR], 2020). Since the emergence of social media, text from such platforms has also been routinely mined directly without any need for physical contact with the participants (Dimond et al., 2012; Robertson, 2017). More technologically capable researchers have also designed, developed, or adopted other tools to analyze online data (e.g., Infovigil for Twitter data by Chew & Eysenbach, 2010). In addition, systematic literature reviews or conceptual papers are also pursued without the need for fieldwork.

Archibald et al (2019) categorize challenges associated with remote data collection into three aspects: ethical, practical, and interactional. Ethical challenges include privacy concerns, data security (Brown, Giguere, et al., 2018), and over-analysis (e.g., “moving close to capture details … encountering the most severe consequence of ‘death by data’—such as magnifying events that may not be significant to participants with regards to selecting what to videotape,” Blikstad-Balas, 2017, p. 511). Loss of contextual data if participants join only with audio in online or in phone-based conversations (Communications for Research, 2021) and visibility of participant due to quality of camera or lighting (Lobe et al., 2020) impact both the interactional and practical aspects (Drabble et al., 2016; Holt, 2010; Smith, 2005). Other interactional challenges arise from reduced control of researchers over the interaction, including distraction within participants’ environment (e.g., other applications or sites, interruptions from phone calls or visits from others, Lobe et al., 2020), late arrival of participants’ early leavers, unexpected no-shows, or late cancellations easy exit (and thus loss) of participants (Daniels et al., 2019). Other practical challenges include bandwidth, network connectivity, and coverage, “high message costs; poor data access” (Brown, Giguere, et al., 2018, p. 78). If participants use a phone to access research instruments, other limitations such as screen display, phone memory, battery life, etc might also apply (Cyprian et al., 2016). To overcome some of these challenges, Daniels et al. (2019) recommend over-sampling to mitigate dropout, participant unavailability, and poor quality of some collected data. They further recommend securing technical assistance (e.g., offering test calls to develop rapport and test technical setup) to help ease potential stress related to online participation. Participants can be asked to mute when not speaking or blur the background to aid privacy protection. For groups, Daniels et al. (2019) and Lobe et al. (2020) recommend breaking groups into smaller ones or replaced with one-on-one sessions as online group discussion is generally more challenging to manage. Daniels et al. (2019) also recommend pre-determining minimum group size if a focus group is used.

### Innovation in Data Collection During COVID-19

Since the onset of COVID-19, research design has often been reframed or adjusted to remote or other innovative data collection methods to maintain social distance (Lobe et al., 2020). Similar to the time before COVID-19, online data collection become widely used (Lobe et al., 2020) through tablets (Dickinson et al., 2019), personal computers, smartphones, and regular phones (Bratcher, 2020; Chew & Eysenbach, 2010; Lobe et al., 2020).

Upon reflection on challenges and resolutions to remote data collection during COVID-19, Bratcher (2020) highlights the importance to make allowance for more time, both for preparation of fieldwork and data collection. Delays should be expected. Sy et al. (2020) further highlight the importance of assessing the understanding, abilities, and aptitude of participants to use technological applications or devices before embarking on any adaptation to research tools or platforms. Due to uneven access to the internet or other remote devices of infrastructure, Sy et al. (2020) also caution against potential sampling biases. Hensen et al. (2021) similarly caution against rushed planning and biased sampling (e.g., over-reliance on non-probability and convenience sampling) at the risk of producing biased or misleading findings.

## Method

This article reports a self-study of the two authors’ experience, observation, critical reflection, and dialogue regarding data collection during COVID-19. Thus, interprevism paradigm was adopted. Nickerson (2022) describes interprevism paradigm as an approach which helps to understanding the motivations, beliefs, and reasoning of a person(s) in a social issue such as COVID-19 in this context. Interpretivism research paradigm is crucial to the decoding of the meaning of collected data with regards to a phenomenon. Meanwhile, self-study focuses on practice with an aim to improve practice through learning (Idris et al., 2021; Samaras, 2011). It incorporates recollection of one’s practice and reflection on factors that might have impacted practice (e.g., identity), but goes beyond reflexivity. For one, self-study is a collective reflection as the method requires collaborative inquiry, dialogue, and feedback with critical friends who interrogate and assist in deeper reflection. According to one of the main pioneers of this methodology Samaras (2011), “critical friends are trusted colleagues who seek support and validation of their research to gain new perspectives in understanding and reframing of their interpretations” (p. 5). In this study, the two authors act as critical friends to each other. Another distinctive feature of self-study is its systematic incorporation of literature where reflection is constantly compared and contrasted with literature to derive meaning. In this sense, it is a “disciplined and systematic inquiry” (Loughran, 2007, p. 19).

Self-study researchers clearly identify the problem or focus; provide a detailed description of the situated practice; explain the self-study method and why it was chosen; describe the multiple data sources; provide a clear explanation of any alternative forms of data employed…establish trustworthiness; include a thorough and transparent data trail; and offer a discussion of the findings to themselves, to others and to the field (Samaras, 2011, p. 14)

In theory, self-study can be used for any professional practice. In practice, however, the majority of the publications using this methodology are on teaching practice or teacher education, including those for (teaching) leadership practice (Frick & Riley, 2010). Besides self-studying teaching, Thompson (2004) adopts it to study faculty workload, Samaras et al. (2012) self-study a professional organization. Rarely has it been experimented with by researchers to self-study their research practices. Among the few that do, Schulte (2001) uses it to self-study supervision practice; Pinnegar and Quiles-Fernández (2018) explored self-study beginning researchers’ relationships with research participants; Arditti et al. (2010) focus on the role of emotions in fieldwork; while Makaiau et al. (2015) use it to study international research collaboration. Meanwhile, following the search online, no research has used this methodology to examine the data collection process or COVID-19’s impact on this process. This form of self-study can be described as duoethnography considering the involvement of two researchers. Duoethnography was adopted because it is a creative approach to qualitative research (Kinnear & Ruggunan, 2019). According to Fitzpatrick and Farquhar (2018), Ellis et al. (2011), it is a form of collaborative autoethnography. Meanwhile, autoethnography is a report of a researcher’s personal experience(s) targeted at communicating understanding about a specific societal phenomenon (Kinnear & Ruggunan, 2019). Duoethnography is relatively new and allows for storytelling of two researchers from their different points of view over a given phenomenon (Given, 2008). In this study, the two researchers present their point of view with regards to data collection for research during COVID-19 pandemic. Given (2008) further identifies four tenets that must be taking into cognisance when adopting the duoethnography. These are:

1. Ensuring that the methodology remains open to allow for flexibility. This allows the researchers to adapt the method to their unique circumstances ensuring that they are guided by the basic tenets. This made it possible for the two researchers to adopt this method in this study.
2. Each researcher’s voice must be clearly presented. Thus, the two researchers ensured that their voices were clearly presented. This was done using “Ç” and “Y,” respectively to represent the researchers.
3. Emphasis is to be on the quest or questioning. The emphasis is not on uncovering meanings rather on creating and transforming them. Given (2008) cautions that researchers are to be careful not to present themselves as the hero or victim. In the context of this study, the two researchers ensured that emphasis was on questioning. Thus, there were series of back and forth as each researcher questioned the other, their reported practice and research.
4. Reporting the differences between researchers is to be encouraged. The differences in the points of view of researchers are considered as strength. In this study, attempts are made to present the differences between the two researchers.

The researchers concur with the literature that empirical studies are not the only type of research. Similar to the practices adopted by some scholars in terms of writing without empirical data, C employed review methods for three of his publications since the start of the pandemic although he seldomly did so before COVID-19. For Y, conceptual or review paper is the genre Y used before COVID-19, so she continued with this tradition and also worked on a few writing projects that don’t involve data collection during this period. Neither experimented on participants’ self-collecting data, secondary data analysis, data mining from big data during this period, however. As this article is mainly about data collection in empirical studies, it is important to give a brief description of the research projects reflected in this article. For C, this mainly refers to two projects (C1 and C2, both team projects). C1 used a mixed-method approach to investigate students’ adjustments to online learning in two tertiary institutions in South Africa. Data were collected from both the students (200 per institution) and academic and support staff (four each) from four and six faculties respectively. C2 surveyed the experiences of international students in tertiary institutions during COVID-19. All 26 institutions in South Africa were planned for this study, although only six institutions eventually participated with a total of 41 responses. For Y, the studies reflected in this article include two institutional projects. Y1 is a team project of university experiences of underprivileged students in 2 faculties in one institution, involving secondary analysis of institutional data and interviews with 6 academics, 4 support staff, 6 administrators, and 32 students. Y2 surveyed and interviewed students’ experiences and views of using Whatsapp for teaching and learning during COVID-19 in one institution. In Y2, all students enrolled in two modules (about 700 students) were surveyed first (92 responses were returned). Among these 17 indicated a willingness to be interviewed and 10 interviews were eventually realized. Y also reflects on three projects she oversaw as supervisor during COVID-19: y1 interviewed six school principals about their prioritizing experience during COVID-19; y2 collected and analyzed teachers’ perceptions about stress and stress management during COVID-19 using mix method (all teachers in three schools, 24 survey responses were returned and three were interviewed); y3 interviewed teachers and their Head of Department for experience and management of out-of-filed teaching (three schools, two teachers, and one HoD in each school).

Most of these topics are related to the pandemic, similar to the broad trend observed during COVID-19. The only exception is y3 who initially planned to research related to ICT teaching, but settled down on out-of-field teaching as the topic is closer to his own experience and interest. Both C1 and Y2 are the direct result of the change to online emergency teaching. C2 is about students’ experience during COVID-19. Both y1 and y2’s topics were decided during a further brainstorming on topics and research questions although both students had originally signed up for other topics (not related to COVID-19). For Y1, although the project commenced before COVID-19 and is part of a larger multi-institutional study, the impact of COVID-19 was added as one example of changes in intuitional culture after fieldwork started. This shows the different ways COVID-19 impacts the research topic.

### Researcher Identity and Impact on Research

As identity does not only determine what one values, what profession one pursues, what goal one sets, but also how one pursues one’s goals (Akosah-Twumas et al., 2018), self-study invites retrospection of how one’s professional identity impacts on his or her professional practice (Samaras, 2011). Reflecting on identity also aids crystalizing and shaping one’s sense of identity, in turn one’s professional trajectory. Harvey (2013) suggests that foregrounding a researcher’s identity enhances research transparency and interpretation of research results. Interrogating researcher identity also enhances the level of analytical criticality as it aids researchers to be aware of their assumptions of various research decisions (Harvey, 2013; Scaratti et al., 2021) including research questions and theoretical frameworks choices, methodological orientation, and methods of analysis (Parker, 2020).

For the two authors of this article, both are experienced researchers although this is the first self-study for both. It is also the first time the two collaborate on research. C has achieved an *h*-index of 10 and i10-index 11 since he completed his PhD 4 years ago. He values large data sets and considers himself more of a quantitative researcher although he also conducts qualitative enquires. C used to teach and supervise before COVID-19. More recently, C has started a research associate position where his primary responsibilities are research and supervision. C’s main research area is (in)equality in education. Much of his research is institutional studies on topics such as educational changes, rural education, and technology incorporation in teaching and learning. In addition, C also researches and writes about entrepreneurial education and higher education. C considers self-reflection a key component in his professional growth, thus often takes out time to think and reflect on the past in his present dealings in research and academic endeavours. C is of African origin. He has schooled, worked, and conducted research in rural areas, which inform his research interest and practices. Y completed her PhD about 14 years ago. She is generally more oriented toward the qualitative approach for her empirical works, although she jokingly claims that she is usually qualitative in a quantitative team and quantitative in a qualitative team. Y teaches, research, and supervises (before and during COVID-19) but considers research most fundamental to her professional identity. Y routinely incorporates reflection in her teaching (e.g., encourages or includes reflection as assignments or module outcomes) and applies reflectivity in her research, professional development training, and administrative work with continuous tinkering and reflection on ways for further improvement. Self-claimed to be a multipotentialite, Y researches on and supervise a wide range of topics such as education policy, leadership and management, research ethics, cultural studies, bilingual education, higher education studies, China, and ICT. Chinese in origin, pragmatism, and resulted task orientation are key approaches in Y’s professional and personal pursuit.

### Sampling Strategy and Participant Recruitment

The sampling strategy is a practical tool to extract a smaller set from a large population one as a single research study usually doesn’t include all study populations (Landreneau & Creek 2009). One key consideration in selecting a sample strategy is to balance feasibility (e.g., achieving an acceptable response rate; Brown, Low, et al., 2018) and minimizing sampling bias to ensure research quality. This is particularly important in quantitative studies where a representative sample is essential to generalize the finding or draw inferences from the sample to the population (Bhandari, 2021). Sampling bias, occurring “when some members of a population are systematically more likely to be selected in a sample than others” (Bhandari, 2021, p. 1), distorts the results of a study (Galdas, 2017, p. 1). To avoid bias, convenience sampling is generally to be avoided (Bhandari, 2021). Instead, the adoption of probability sampling (including simple random, stratified random, cluster, and systematic sampling) to give qualified participants equal opportunity to be selected is advised (Creswell, 2014; Kumar, 2019). For qualitative studies, generalization is often a less important concern while the ability to provide in-depth information is often deemed more imperative (Creswell, 2014; Kumar, 2019). In these studies, the researcher selects participants who fulfill certain pre-determined criteria or categories and are expected to be able to provide rich data. This means that non-probability sampling including convenience, quota, purposive, and snowball methods are often accepted in qualitative studies (Creswell, 2014; Kumar, 2019) despite their greater likelihood to incur bias (Skowronek & Duerr, 2009). Instead, Greene and McClintock (1985), Tuckett (2004), as well as Oppong (2013) and Hassmain (2020) all suggest the inclusion of different data collection techniques or employing a mixed-method approach to allow further interrogation of similarities or differences in data to ensure data and research quality.

Sampling strategies also need to be feasible and realistic, however. No matter how great a sampling strategy appears on paper, if insufficient or inappropriate participants can be realized, recruited, or secured, the collected data and findings will be unable to lend to a planned level of generalization or simply become inaccurate (Kumar, 2019; Vasileiou et al., 2018). As participants’ interest changes (e.g., become more attentive to other more urgent matters) during crises, locating, accessing recruiting willing participants often becomes more difficult (Bratcher, 2020; Meier, 2014; Qadir, 2016) and one key concern when selecting sampling strategies during these times. Although online data collection has the potential to reach a larger population, certain participants are more likely to be systematically excluded due to network and other infrastructural challenges, impacting sampling bias (Carter et al., 2021). In these circumstances, researchers often set minimum requirement for sampling (e.g., sample size or participant criteria), then cast net as widely as possible to reach participants who fit those minimum requirements (Martínez-Mesa et al., 2014, 2016). Personal networks, social media platforms (personal or institutional accounts) as well as other media outlets (physical or online, including google advertisements), are sometimes used to advertise and reach participants (Ali Shahmir et al., 2020; Zhong et al., 2020). Even where researchers may desire to adopt other sampling strategies, convenience sampling becomes practiced more often. Snowball, a sampling strategy often used for sensitive topics or hard-to-reach populations, for example, sexuality or drag dealers, is also used more often (Archibald et al., 2019).

The difficulty of accessing participants and its implication for sampling strategy and execution are observed in all studies studied here. The potential of remote data collection to reach a larger participants pool was not observed in any of the studies. Non-probability sampling (purposive, quota, convenience, and snowball) was widely used, even for quantitative studies. For instance, convenience sampling was involved in selecting the institutions and the participants in C1 *after* the initial criteria is fulfilled (one rural and one semi-urban institution and students in tertiary institutions for more than 1 year). The same difficulty also leads to more relaxed criteria for participant selection or the lesser stringent screening in some cases. In Y1, one preferred criterion for some categories of participants to have worked in the institution for more than 2 years was relaxed to those recommended by HoDs or directors. Participant replacement also occurred in Y1 when the staff for a certain category agreed but didn’t show up after arranging the meeting a few times. For C2, Y2, and y2, the *population* was determined via convenience consideration: students enrolled in two undergraduate modules whose lecturers are known to Y in Y2, which Y knew that she could ask the lecturers to administer research instrument; participating schools in y2 were in the same area as the school y2 teaches herself where she knew some school principals and teachers; the schools y3 chose were all recommended by y3’s own principal. For some studies (e.g., C1, Y1, and y1), when *population* or *samples* in terms of targeted categories and quota of the institutions and participants was pre-determined, criteria were determined in conjunction with accessibility consideration, for example, schools or participants’ categories involved in y2 and y3.

Another key observation from our experience is the importance of approaching and contacting the right people for increasing the chances of securing respondents. Besides impacting the decision in terms of which institutions or participants to include, people previously known to the researcher also played an important role in facilitating participants’ access in all studies besides providing reference in snowball sampling. This is in congruence with the work of Mack et al. (2005). Sometimes this middle person is also a potential gatekeeper, for example, a principal or district officer in the case of schools. IT advice of not opening emails from unfamiliar sources, clicking on a link or opening an attachment to minimize spam might have potentially accelerated this importance of facilitating familiar sources (E. J. Williams et al., 2018). In C1, two people from the two institutions were the point of contact throughout data collection, one even acted as the research assistant to visit participants and help secure questionnaire (hardcopy) distribution. This facilitation was crucial even though participant details (emails or contact numbers) were provided by the IT office in respective institutions following due protocols, and emails or WhatsApp messages and reminders had followed initial contact. In C2, one leader in an international student association in one institution offered to administer the survey to international students in his institution as well as similar associations in other institutions. In both of these cases, the familiar middle person helps to ease and fast track data collection, similar to the findings from Geertz (1973) and Sanghera and Bjorkert (2008). The middle person also helped with certain categories of participants who were harder to reach, including academic participants (compared to students although both were off-campus in Y1 and C1) and senior management in schools (as compared to educators or HoDs in schools in y2 and y3). Again, the prior network and the middle person often came to the rescue. The researcher in y1 was a school principal herself and her participants were from her prior contacts from principals’ conferences. In all cases, insider knowledge, a sense of familiarity via a person already known to the participants has helped tremendously. This referral seems to be particularly important during a crisis when participants are overwhelmed with unexpected or additional responsibilities, stressed, and anxious (Kennedy-Shaffer et al., 2021). Snowball, from those who have already responded to combated low survey responses (Creswell, 2014; Kumar, 2019), was also used more during this period: to reach the targeted sample size (C1) and increase realized sample in C2 where snowball added 17 participants (or 41% to total sample size). In Y1 when academic participants weren’t responding to invitations to participate, students and administrators were asked to recommend lecturers.

This however highlights the importance of further discussions on sampling bias from referrers or middle person’s influence (Simkus, 2022). Holmes (2020) further cautions about the possibility of the middle person jeopardizing data quality when respondents become uncomfortable or unwilling to divulge certain information. Besides the possibility that the middle person only recommends or refers to certain types of participants, this also calls for further discussion on the potential limitation of generalizability (Robinson, 2020; Kirchherr & Charles, 2018; Morgan, 2008).

A low response rate also meant a longer duration for data collection, similar to Bratcher (2020) and Sy et al.’s (2020) observations. This is caused both by delays in locating potential participants and delays in reaching an agreement on the logistics of administering the research instrument. Although these challenges existed before COVID-19, more limited channels of access (e.g., no longer possible to knock on an office door or call an office number), change of priority (e.g., sudden and expedited transition to teaching online), the uncertainty of the situation, general confusion and in some cases participant health, and aggravated the scale of the challenges. Despite potential time saving through digital data capturing and automatic transcription (e.g., in the case of the transcription function from Microsoft Teams), these saves in time couldn’t compensate for the prolonged data collection time.

Additional observations include:

- A clear difference emerges regarding participant access and data collection duration due to limitations or the possibility of physical access. All studies except C1 were conducted completely online, but at institution 2 in C1, COVID-19 regulation was relaxed during data collection when students were briefly back to campus residences due to complaints of data and network issues and in preparation for exams (although their lectures were still online). C1 took advantage of this period and distributed hardcopy surveys. This compressed data collection from two and a half months in another participating institution to 2 weeks in this institution to reach the same amount of returned surveys.
- All studies were prone to potential bias due to technological access or skills but it was hard to determine the scale of this bias and its impact on data quality, wanting of control groups.

The above does not indicate achieving sampling size taking precedent over consideration for bias or appropriate sampling strategy, but practical consideration does emerge much more salient during a crisis. This results in a much harder decision on the potential trade-off between rigorous sampling and feasibility (Bhandari, 2021). For all the quantitative studies reflected here, triangulation of data from different sources and instruments emerges as the primary way to minimize bias and ensure data validity, concurring with scholars’ suggestions (Greene & McClintock, 1985; Hassmain, 2020; Oppong, 2013; Tuckett, 2004). Other mixed-method studies also enjoyed similar benefits. The challenges of pure quantitative studies are expected to be the largest, but there are too few such studies included here for further and deeper reflection.

### Select Data Collection Platforms and Instrument

Data collection platforms and instruments also impact the quality of data collected. Both are also prone to challenges brought about by participant accessibility (Sarfin, 2021). Besides common challenges related to the platforms (discussed in the literature review), there are also challenges specifically related to instrument designing. These challenges include recall bias (e.g., recall viability or accuracy; Mahtani et al., 2018), social desirability, *or conformity bias* (Great Brook, 2022), inadequate attention (due to research fatigue or participant annoyance or inadequate attention due to whatever reasons) (Kabir, 2016), presence of leading, misleading, or loaded questions, etc. (Allen, 2017). Quantitative instruments are prone to additional challenges lacking interaction and opportunities to clarify or explain (Andrade, 2020). As a remedy, Bhandari (2022) suggests asking specific questions, one question at a time, questions are kept simple, and the questionnaire is kept short. According to Ball (2019), questionnaire validation should be a major step to ensure that “the questions capture the anticipated data and are not interpreted differently by researchers and participants (p. 415).” With the qualitative instruments, challenges are usually related more to the *administration* of the instruments, for example, to “entice” the participant to speak more freely and more fully while not letting one’s subjectivity get in the way (Khankeh et al., 2015). Researchers’ research skills (ability to pick up small nuances in participants’ response, probing etc.), as well as personal and social skills (e.g., make participant feel ease), becomes more critical (Austin & Sutton, 2014). This is another area of balance researchers need to judge and decide: namely that between the ability to generate sufficient, reliable, and high-quality data and those related to feasibility.

Building rapport is generally seen as one crucial step that has the potential to contribute to quality data through relationship building (Keiling, 2021). This is particularly crucial in qualitative studies where relaxed and trusting participants speak more fully and more truthfully (Keiling, 2021; Bell et al., 2016) and produce more rich and in-depth data (Guillemin & Heggen, 2009). Koh (2022) suggests the researchers should dress appropriately (not over or underdress), be accommodative, take cues on body language, showing interest in the participants as ways to build rapport. Ameyo (2016) offers further tips such as beginning and continuing the conversation in a friendly manner, listening with rapt attention, giving full attention during the conversation. This indicates that rapport can and should happen *during* instead of *before* data collection as literature often suggests (Bell et al., 2016). In other words, rapport can happen beyond seeking clarity in the pronunciation of the name of the interviewee, preferred name, small talks related to the research topic among others (Bondaug-Winn, 2021). Building rapport can also be completed remotely, for example, through phone calls (Hensen et al., 2021; Reñosa, et al., 2021; Salmons, 2012) or typed words (sending and receiving messages) instead of having to be face to face. The key is to show interest and respect in terms of participants’ knowledge and willingness to share.

There is also limited literature on the degree of ease of establishing rapport during remote data collection or the difference in building rapport between face-to-face and remote data collection. COVID-19 has induced anxiety causes additional confusion and exacerbates attention span and data quality, but direct discussion since the onset of the pandemic is scarce. Literature before COVID-19 indicates that while some report no difficulty in establishing rapport and free expression with strangers not hampered by the remote environment (Archibald et al., 2019), some report participants being more responsive and building rapport more quickly online (Deakin & Wakefield, 2014; Tuttas, 2015), others report it more challenging in a remote environment (Cater, 2011).

Both authors have limited experience collecting data online before COVID-19. For C, he used to walk to the lecture halls or student residences to explain his research and administer the instrument before COVID-19. “Small” talks on issues such as academic pursuit, well-being, etc were one way C built rapport before bringing up discussions about his research. Among Y’s studies before COVID-19, emails together with phone calls were routinely used to contact potential participants initially. Among the few quantitative studies she was involved in, she seldom directly administered hard copy surveys although she had administered online surveys where rapport features little in the execution of the data collection. Instead, the focus was often more on survey design itself where questions were designed through literature, existing instruments, consultation with statisticians and instrument piloting. For qualitative studies, rapport was usually done through ice breakers and small talks at the onset of the interviews, usually not before or on a separate occasion. Y however usually pays great attention to listening attentively and following up on questions during the interviews.

During COVID-19, online platforms became the primary channel for data collection for both authors (except institution 2 in C1). The majority of participants for C’s studies were accessed through phone calls, WhatsApp, or emails. Similar to the experience by Bratcher (2020), Lobe, Morgan and Hoffman (2020), and Ratislavová and Ratislav (2014), the majority of C’s participants preferred emails exchange (instead of other online interview platforms) during COVID-19. More specifically, open-ended questions in email replaced interviews where the responses were generally adequate therefore no further probing was deemed necessary. For Y1 and Y2, Microsoft Teams was the default interview platform, because Teams was the official platform adopted by the institution since the onset of the pandemic although almost all online interviews were conducted audio-only, due to concerns of bandwidth and data cost if the video would be used. This institutional endorsement and Team’s transcription function increased its popularity where the usage of Zooms never took off in any of Y’s studies (probably also because of its 45 m duration limit for its free version). Emails were still used to reach the participants and secure consent, but were never used as a data collection tool. For surveys, google form emerges as the most popular platform due to its unlimited question and zero cost. For the projects Y supervised, data collection platforms ranged from Whatsapp voice note recording, Whatsapp messages, Zoom, Teams, face to face (when participants were comfortable) for interviews and Google forms for surveys.

For respondents previously known to C, C continued his “small” talks before administering the research instrument. In congruence with the work of Salmons (2012), when C sent an introductory message through WhatsApp or email to those previously unknown to him, he stated the name of the referrer to gain the trust of the participants. But it felt strange to do so with someone unknown, so for both questionnaire and email interviews, C would often go straight to the point, explaining after greeting, “sacrificing” the informal rapport building. For Y, similar practice regarding rapport building (or the lack of it) continued during COVID-19.

Our main observation regarding COVID-19’s impact on the quality of data collection is manifested in the following aspects:

- Interruptions. Interruptions happen in face-to-face interviews (e.g., others walk in), but much more often online (from a crying child, to others walking into the room, receiving phone calls etc). A number of interviews in Y1 and Y2 were completed in more than one session; on one occasion (Y1), the interview was completed in three sessions.
- Interview and recording length and quality vary as internet speed and researcher skill vary: many transcripts featured “can you hear” often; some transcripts show much more probes than others (especially in Y1 where field workers were involved in data collection). For occasional face-to-face interviews, masks sometimes obscured recording quality.
- Some participants seem rushed in the interviews or provided very brief answers only, although this was not universal and others’ responses were lengthy and rich (one interview scheduled for 1 hour lasted for 2 hours as the participant continued talking). In C2 where open-ended questions were asked via email, many participants gave brief answers.
- The use of emails in C2 and audio-only for interviews did not allow for observation of participants’ facial reactions or body language. This might have resulted in the loss of data although upon further reflection, we realize that the content of the conversation (rather than context) has been the primary target of analysis before and during COVID-19.

Interruption likely impacts participants’ attention during the interviews although skilled interviewers can still bring participants back on track. The length or richness of the responses is likely caused by a combination of factors including participants’ characteristics (e.g., some participants might be by nature more talkative or are interested in the topic and want to contribute more), research skills, the possibility that at home is more comfortable (and therefore less rushed), or consideration for data cost or bundle being depleted (therefore more rushed). Reliance on email as a substitute for interviews also put greater emphasis on the quality of instrument design. Although only a small difference can be detected in terms of how one establishes and how easily it is to establish rapport during COVID-19, we do discern a greater need for researchers’ research skills (including those related to rapport building, instrument designing and instrument administration), in congruence with Salmons’ (2012) and Tremblay et al.’s (2021) conclusion.

### Ethical Considerations and Practices

Although ethics “should be addressed at all stages of the data lifecycle” (Tarrant et al., 2020, p. 3), it is particularly important during data collection. It needs to be carefully planned and continuously evaluated (Newman et al., 2021). Besides ethics related to sampling fairness, ethics principles also often refer to respect, voluntary participation (usually through informed consent), privacy and confidentiality (e.g., data protection, storage, and accessibility), and beneficence and no harm (Bhandari, 2021; Vanclay et al., 2013). In addition to potential benefits from the research should outweigh risks (Reyes, 2020, p. 29), research ethics also needs to balance between consideration of all ethical principles and scientific merit that justifies the need to conduct the research or collect data (Weinbaum et al., 2019). Ethics clearance is routinely required before data collection where the ethics committee judges whether the researchers have adequately considered or addressed the ethics principles and scientific merit. After ethics approval, informed consent is another critical tool during participants’ engagement where research aims and objectives, the expectation for the participants (e.g., what participation entails, etc.), and other aspects of ethics (e.g., confidentiality and anonymity) are explained. Informed consent is usually obtained through participants’ signatures on the form, prior to data collection.

Upholding ethical practices is paramount regardless of the situation (Peterman et al., 2020). “A crisis is not a time to throw ethics out of the window” (Tarrant et al., 2020, p. 2). The fundamental ethical considerations in remote data collection are no different from those in face-to-face contexts (Lobe et al., 2020; Newman, et al., 2021), although a crisis might post additional ethical challenges (Hsu et al., 2021; Kim & Grady, 2020), such as adhere to health protocols or regulation. Date security or privacy for remote data collection refers to the permanency of online storage and the possibility of local storage (e.g., record from own device or recording link is provided to all participants in Skype, Hassmain, 2020). The balance between scientific merit and research need comes to the fore even more as participants might have extra work demands and their emotional state might be generally more volatile or vulnerable. Interestingly, remote data collection might tip the power back to the participant as withdrawal is easier, for example, by simply disconnecting (Lobe et al., 2020), muting or disabling video, or blaming the internet connection where participants are much less likely to withdraw once they’ve started with the research instrument, likely due to tacit social norm.

Research ethics were all considered and ethics clearance was obtained before data collection for all studies reflected here. For informed consent, Similar to the practice before COVID-19 where informed consent was usually emailed or provided in advance and additional questions for clarification or debriefing were conducted prior to data collection. However, due to the difficulty of participant access, after many trials to secure their participation, both authors’ temptation during COVIC-19 is to administer the instrument immediately the moment they agree to participate (verbal or written), impacting the rapport building and potentially data quality. As some participants do not have an electronic signature or are not bothered to through the hurdle of completing the consent form, verbal consent was accepted instead of insisting on signing as the ethics application since the onset of COVID-19 has included such adjustment and was approved. Although the ethics committee’s endorsement on this is not uniform (Chatha & Bretz, 2020), this is endorsed by the Human Research Protection Program (HRPP, 2021) and Research Support (n.d., p. 1) who approve an oral or a waiver of signed consent “where time for consent is limited, for example, a chance interaction between researcher and participant” and when there is minimal risk. In addition, participants’ willingness to complete the research instrument is also sometimes accepted as an indication of their consent, although this is not included in ethics clearance and this practice or implication of this practice, features little discussion in the literature.

For data privacy, the software used for online interviews reflected in this study is Teams and Zoom which only allow meeting initiators to record. In terms of the concern that participants’ surroundings might be visible during interviews (Lobe et al., 2020), all participants opted for audio-only (instead of video interviews), likely due to data cost concerns. With regards to the quantitative components, the links to the questionnaires were sent to the potential respondents. The analyzed data were stored in the personal computer of the researchers who are the only ones to access the data.

Both authors also found themselves more sensitive toward participants’ reluctance to participate, forgetfulness in attending agreed-upon meetings or general difficulty of accessing. In the context of C1 when C had to contact the participants online without the use of a middleman, he and his research partner sometimes felt that asking for participation was intruding and not welcoming when the participants were slow to respond, skipped, or were late for appointments, or distracted or rushed during appointments. This concern was less salient in the cases when the middle person was involved and the one step removed from direct interaction with the participants, however. Y also found herself sometimes torn between the urge to follow up (before participants agreed to participate or when meeting days were shifted) and holding it back as she pondered the stressful situation the participants were in. When participants agreed to the interview initially but went quiet later, Y didn’t press further.

Additional ethical consideration relates to cost. Before COVID-19, research costs were mainly incurred only by the researcher in terms of printing the questionnaire, travel for data collection, and data capturing. Except for travel to attend focus groups, participants’ cost was generally limited to time. During COVID-19, however, travel was replaced with data cost in terms of data bundles and airtime and incurred for both the researcher and the participants. This might have exacerbated participants’ reluctance to participate in research during COVID-19 and reinforce a popular notion among the participants that research participation is mainly to assist researchers to complete a study with little immediate benefit to them (K. Yu, 2008a). Participant compensation in this case becomes not only one way to increase the response rate (S. Yu et al., 2017), but a more appropriate ethical decision (Chen et al., 2020). Paying participant or research compensation is a controversial practice usually strongly discouraged by the Research Ethics committee (Head, 2009; Surmiak, 2020; Tyldum, 2012; Zutlevics, 2016) for fear of undue influence. Undue influence is high enough payment (Largent & Lynch, 2017) that “induce prospective participants who otherwise would not enrol to enter studies in which there might be significant risks. The worry is that people with limited resources are more susceptible to inducements to act against their own best interests, or that, worse, they could be targeted for recruitment because they are easier to influence with smaller sums of money” (E. P. Williams & Walter, 2015, p. 1117). Limited discussion, however, exists to differentiate incentives and compensation, undue influence and showing appreciation or goodwill, research of small risks versus significant risks, such practice in different fields, or alternative ethics arguments that *deny* poor participants a chance to make some money from research for (K. Yu, 2008b). Before COVID-19, some researchers provided meals for the participants (or invite participants for meals), offer stationaries, educational materials or uniforms, or vouchers for students, or soap or other goods (Grady, 2005; Ndebele et al., 2008). As per research tradition, all studies reflected here provided participant compensation except Y2 which had a budget where airtime vouchers (for data bundle) were provided after the interviews.

## Conclusion and Recommendations

COVID-19 has disrupted research activities in terms of priority, availability, and attention span (Bratcher, 2020; Mackenzie et al., 2021). Much of these disruptions do not only impact the practicality of data collection, but potentially also data and research quality, through for example sampling bias, “compromised” generalizability and reduced opportunities to explain or clarify. Quantitative studies seem to be harder hit by these challenges; but greater awareness and research skills also become more in demand for qualitative studies during COVID-19. Although avoiding physical data collection has been experimented with before and during this pandemic, this seems to be only feasible to *supplement* research activities rather than completely *replace* the needs for data collection, hence revisiting data collection during a crisis is still valuable. Using duoethnography method for reflecting on research activities, the authors review and reflect on the practices of remote data collection during the pandemic and further revisits additional issues brought about by these practices and concerns. Is the finding of the study shows that although innovative data collection experiments before COVID-19 have demonstrated potential benefits of remote data collection, such potential benefits are easily offset by the much greater difficulties regarding participant access during this crisis. This practical challenge prevails and overshadows other challenges Archibald et al. (2019) identify. This challenge requires more flexibility for the researchers in terms of revising the research topic, adjusting research tools or sampling, considering alternative ways to secure participants or collect data, accommodating (or finding alternatives to) any changes in terms of participants’ willingness, considering alternative forms of consent, and setting aside longer time for complete data collection. All implies researchers’ reduced control of the research process. Data collection during COVID-19 also requires greater researcher skills in terms of finer tuning to participants’ concerns and needs—often completely rely on participants’ self-reporting and without visual cues—and the ability to “entice” them to speak fully and maintain attention to the research topic.

We observe greater conflation of quantitative and qualitative approaches in data collection and echo other scholars’ questions of the adequacy of these labels (Abrams et al., 2021). For example, a study can target a large sample but does not use probability sampling (e.g., C2) or interview data collected through WhatsApp chat or email that resembles more questionnaires than the conventional understanding of interviews. We also note evidence, albeit limited, of the difference between face-to-face and remote mode in terms of ease and duration of data collection.

Among potential strategies that can offset potential threats to data quality, triangulation from different approaches or data sources emerges as one of the most effective and feasible tools. Discussions on the adequacy of this (alone), however, remain limited (Flick, 2018). Building and maintaining rapport also seems to be in greater need to offset more interruptions and participants’ shorter attention span, although we found that more attention should be put on rapport *during* data collection, rather than a mere emphasis on rapport *before* data collection. Rapport also needs to go beyond rhetoric, especially for quantitative studies.

We echo suggestions other scholars have made for remote data collection, including oversampling, the need to budget more time, and paying attention to technical issues or skills (Bratcher, 2020; Daniels et al., 2019). But this article calls for more discussions on several areas that features scarce discussion in literature, including potential rhetoric importance assigned to data collection.

Additionally, following the findings of the study, the authors suggest that even before the advent of any future pandemic, researchers should begin to identify and strategize ways of collecting data remotely in more convenient and appropriate manner. The practices should be such that they are practicable during pandemics or otherwise. This would enable researchers in their conduct of research even during the time of global crisis such as the Covid-19 pandemic.
